# Interactive film scenes for tutor training in problem-based learning (PBL): dealing with difficult situations

**DOI:** 10.1186/1472-6920-10-52

**Published:** 2010-07-06

**Authors:** Hans M Bosse, Soeren Huwendiek, Silvia Skelin, Michael Kirschfink, Christoph Nikendei

**Affiliations:** 1 Department of General Pediatrics, Centre of Child and Adolescent Medicine, Im Neuenheimer Feld 430, 69120 Heidelberg, Germany; 2 Department of Neonatology, Centre of Child and Adolescent Medicine, Im Neuenheimer Feld 153, 69120 Heidelberg, Germany; 3 Institute of Immunology, Im Neuenheimer Feld 305, 69120 Heidelberg, Germany; 4 Department of General Internal and Psychosomatic Medicine, University of Heidelberg Medical Hospital, Im Neuenheimer Feld 410, 69120 Heidelberg, Germany

## Abstract

**Background:**

In problem-based learning (PBL), tutors play an essential role in facilitating and efficiently structuring tutorials to enable students to construct individual cognitive networks, and have a significant impact on students' performance in subsequent assessments. The necessity of elaborate training to fulfil this complex role is undeniable. In the plethora of data on PBL however, little attention has been paid to tutor training which promotes competence in the moderation of specific difficult situations commonly encountered in PBL tutorials.

**Methods:**

Major interactive obstacles arising in PBL tutorials were identified from prior publications. Potential solutions were defined by an expert group. Video clips were produced addressing the tutor's role and providing exemplary solutions. These clips were embedded in a PBL tutor-training course at our medical faculty combining PBL self-experience with a non-medical case. Trainees provided pre- and post-intervention self-efficacy ratings regarding their PBL-related knowledge, skills, and attitudes, as well as their acceptance and the feasibility of integrating the video clips into PBL tutor-training (all items: 100 = completely agree, 0 = don't agree at all).

**Results:**

An interactive online tool for PBL tutor training was developed comprising 18 video clips highlighting difficult situations in PBL tutorials to encourage trainees to develop and formulate their own intervention strategies. In subsequent sequences, potential interventions are presented for the specific scenario, with a concluding discussion which addresses unresolved issues.

The tool was well accepted and considered worth the time spent on it (81.62 ± 16.91; 62.94 ± 16.76). Tutors considered the videos to prepare them well to respond to specific challenges in future tutorials (75.98 ± 19.46). The entire training, which comprised PBL self-experience and video clips as integral elements, improved tutor's self-efficacy with respect to dealing with problematic situations (pre: 36.47 ± 26.25, post: 66.99 ± 21.01; p < .0001) and significantly increased appreciation of PBL as a method (pre: 61.33 ± 24.84, post: 76.20 ± 20.12; p < .0001).

**Conclusions:**

The interactive tool with instructional video clips is designed to broaden the view of future PBL tutors in terms of recognizing specific obstacles to functional group dynamics and developing individual intervention strategies. We show that this tool is well accepted and can be successfully integrated into PBL tutor-training. Free access is provided to the entire tool at http://www.medizinische-fakultaet-hd.uni-heidelberg.de/fileadmin/PBLTutorTraining/player.swf.

## Background

Problem-based learning (PBL) represents a major and complex change in higher educational practice. The sequential approach adopted in PBL structurally reflects the daily work of medical doctors in terms of first defining problems and subsequently applying *procedural *[1] (i.e., differential) diagnostic pathways and/or *explanatory *(i.e., pathophysiological) models to resolve unclear issues [2]. This has led to a widespread integration of PBL in many medical curricula throughout the world.

Effective small-group learning, as found in PBL tutorials, relies on functional group processes and *does not result from simply meeting in a group *[3]. The PBL group must be actively engaged in the learning process for individual cognitive networks to be constructed. Apart from providing a conductive learning setting, the benefits of PBL groups lie in their supporting students' socialization into a new and unfamiliar academic environment and their promotion of personal development with respect to tolerance and patience [4]. PBL further offers many benefits to the tutors including personal development as a result of facilitating such learning processes in adult education [5].

A large part of the responsibility for the performance and outcome of a PBL tutorial lies with the tutor, who facilitates students' learning processes and efficiently structures the tutorials [6]. The tutor's ability to effectively facilitate spirited, creative, and effective learning processes is appreciated by students [7]. The tutor strongly influences group functioning and the time spent on self-study activities, both of which have a significant impact the quality of the results achieved by the group within the tutorial, students' achievements in ensuing assessments, and their future interest in the subject matter at hand [8-10]. Furthermore, PBL tutors serve as clinical role models to a greater extent than applies to lecturers in traditional medical programmes [11].

To fulfil these role expectations, tutors will need to be competent in both the respective medical field [12-15] and the methodological approaches required for effective moderation [8,16] in order to enable learners to actively engage in their individual learning process. Both aspects must be balanced by the tutors; compared to non-expert tutors, particularly knowledgeable tutors are tempted to intervene unnecessarily often in PBL discussions while non-expert tutors may not be able to sufficiently challenge students' level of understanding [17].

In dysfunctional PBL groups, disruptions occur in the active and creative processes which are necessary for learning [18,19]. The resulting tension or anxiety hinders learning [20]. Little research has examined specific situations upsetting this functional process in PBL groups. General problems arising in a PBL tutorial relate to problems identified either by faculty developers [21,22], tutors [23], students [7,24,25] or both students and tutors [26,27]. Hendry et al. [27] summarized and ranked the most common group problems addressed in the aforementioned studies as perceived by both students and tutors. Tutors' interventions were seen to be least effective with respect to dealing with *lack of commitment*, *lateness of individuals*, *quiet *or *dominant students*, and *bullying *or *disparaging of students*.

To help tutors become more effective in assuming their complex role in PBL tutorials, training programmes are required which facilitate tutors' reflection upon their own development as teachers [28,29], address their perceptions for critical situations in PBL tutorials, and allow them to practice their intervention strategies. The plethora of data on tutor training primarily focuses on tutors' general moderation techniques [8,17,30]. In his article on PBL, Azer [31] presents a set of challenges faced by PBL tutors as well as *12 tips for successful group facilitation*.

With the aim of transferring these general recommendations to specific, predefined group problems, we developed a set of video clips. In this report, we present an interactive online training tool which incorporates these clips and exemplary intervention strategies for dealing with obstacles arising in tutorials. The tool is designed to enhance tutors' awareness of successful group work, and it is our aim to promote use of the tool within the medical education community. We describe the integration of this tool into PBL tutor-training courses, as practiced at our medical faculty over the past six years. We present data on trainees' acceptance and feasibility ratings of the tool as well as their pre- and post-intervention self-efficacy ratings with regard to their PBL-related knowledge and skills, as well as their ratings of attitudes towards the method. We discuss the added value and the potential of the video clips when integrated into PBL tutor-training sessions.

## Methods

### An explanatory note on the methodological background

The method of problem-based learning (PBL) is based on a sequence of predefined steps which are to be followed in order to resolve a predefined problem. Since being introduced to the field of medical education, the precise definition of these steps has been subject to slight variation from institution to institution. In this study, we refer to the commonly accepted seven sequential steps of PBL (case presentation, problem definition, brainstorming, generating hypotheses, defining learning goals, self study and synthesis) in accordance to the taxonomy by Schmidt [32] and Barrows [33]. In addition, we conclude sessions with a regular feedback [34].

### Definition of issues addressed in the video clips

A literature search was performed to identify problems commonly arising in PBL tutorials. Additional major obstacles to a PBL tutorial were defined by an expert group of five tutors with solid experience in PBL and curriculum design. A total of 14 commonly encountered difficult situations in PBL tutorials which potentially necessitate intervention were identified [24,27]. For each situation, possible intervention strategies were defined. In the case of two situations, two different intervention options were considered adequate. In addition, *getting a group started *and *ending a tutorial *were considered to constitute critical elements for successful tutoring and were therefore included in our series of video clips.

Tutors with solid experience in PBL, curriculum design, and devising screenplays subsequently compiled detailed scripts for each of the resulting 18 specific situations. The scripts included specific potential interventions.

### Writing of film screenplays

A screenplay was developed by the expert group for each of the 18 film clips on the basis of the drafted scripts. A screenplay was outlined with roles for group members and the tutor including intended potential interventions to be conducted by the tutor. The employed underlying case was a non-medical case which we successfully employ in our PBL tutor-training sessions [2].

For each video clip, arguments for (*pro*) and against (*con*) the specific intervention as well as a *solution *are discussed. The *task for the tutor *in the specific setting is outlined in the text.

### Shooting and web-based posting of films

**Figure 1:**
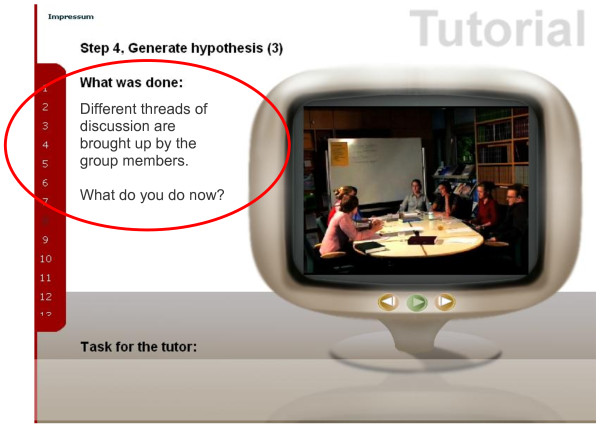
**Exemplary illustration of a sequence - assignment**. The 18 clips can be individually selected for viewing from the play list that opens on the left-hand side. A screen for viewing of the sequences is found on the right-hand side. The buttons allow playing or replaying the respective sequence, or users may proceed to the next sequence. After playing the initial sequence of the video clip, a short summary is displayed on the left-hand side; the tutor is then asked *What do you do now? *(red circle). The buttons allow the viewer to replay the sequence or to continue to the next clip.

**Figure 2:**
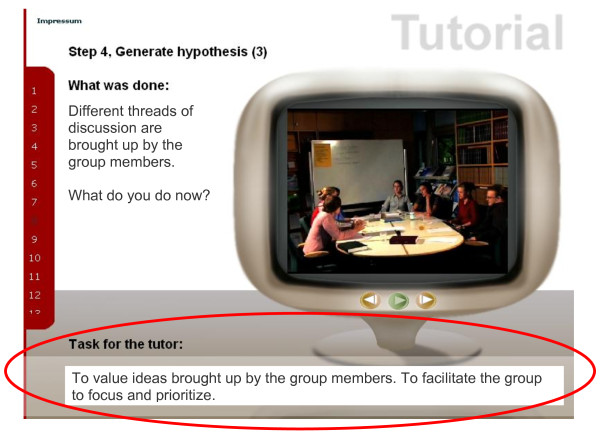
**Exemplary illustration of a sequence - summary**. Following the second sequence in which a potential intervention is illustrated, a short summary of the tutor's task in this sequence is presented (red circle). The buttons allow the viewer to replay the sequence or to continue to the next clip.

The video clips were filmed with volunteering students familiar with PBL as actors which closely followed the respective screenplay. The video clips were processed in an AVI format with final cut. The sequential alignment of film-sequences and script was structured in XML and the video player was realized in Adobe Flash. Therefore an Adobe Flash Player is required to play the videos. For better Flash compatibility the clips were converted to the FLV format. The tool comprising the video clips is available on the web pages of this journal at http://www.medizinische-fakultaet-hd.uni-heidelberg.de/fileadmin/PBLTutorTraining/player.swf with free access and free download (Figure 1, Figure 2).

### Integration of the films into PBL tutor-training courses at our faculty

Regular PBL tutor-training courses for staff of the Heidelberg Medical Faculty include an introductory seminar, experiencing a PBL tutorial as participant using a non-medical case [2], and two-hour interactive training using the presented online tool in its German version, followed by a voluntary post-training peer consulting programme. In the interactive training with integrated video-clips, trainees interactively work on each scenario by first identifying the challenge of the respective critical situation and then, in a second step, developing individual intervention strategies. Trainers in our training courses have substantial experience in PBL tutor-training and curriculum-development.

### Acceptance, feasibility, and self-efficacy ratings

A total of 109 staff of Heidelberg Medical Faculty were trained in three consecutive PBL tutor-training courses in the manner described above. Acceptance and feasibility of the training tool as well as the perceived effect of the course were assessed using a questionnaire previously developed by our group [2].

To assess the perceived effect of the course a questionnaire was administered prior to and after the intervention. The questionnaire comprised items rating the pre- and post-intervention self-efficacy regarding PBL-related knowledge (*I have understood the steps of PBL*; *I have understood the role of the tutor in PBL*) and skills (*I feel well prepared as a PBL tutor; I feel confident facing problematic situations as a tutor*), as well as rating attitudes (*I consider PBL to be a didactically valuable method*). The ratings for these five positively worded items were made using visual analogue scales ranging from 0 = completely disagree to 100 = completely agree. Additionally, prior to the intervention participants provided information on their sex, as well as their age and professional experience (both in years; see Table 1).

**Table 1:** Acceptance and feasibility and perceived effect of the training

	Items	pre	post	significance
1	*I have understood the steps of PBL. *	48.80 ± 29.28	81.92 ± 16.00	p < .0001
2	*I have understood the role of the tutor in PBL. *	46.11 ± 28.89	81.16 ± 15.51	p < .0001
3	*I feel well prepared as a PBL tutor*.	34.46 ± 26.76	70.28 ± 20.08	p < .0001
4	*I feel confident facing problematic situations as a tutor*.	36.47 ± 26.25	66.99 ± 21.01	p < .0001
5	*I consider PBL to be a didactically valuable method*.	61.33 ± 24.84	76.20 ± 20.12	p < .0001
6	*The films enhanced my preparedness to respond to the specific challenges of a PBL tutorial*.		75.98 ± 19.46	
7	*The time frame allocated to the films was adequate*.		62.94 ± 16.76	
8	*The overall concept including the films was reasonable*		81.62 ± 16.91	

Results of the self assessment of N = 109 trainees of three consecutive PBL tutor training courses at our faculty. Data were collected pre-post intervention (items 1-5) and post-intervention (items 6-8; all items visual analogue scales from 100 = completely agree to 0 = completely disagree). Significances are given for the pre-post intervention assessment (items 1-5).

After the intervention three additional items were assessed relating to the acceptance and feasibility of the films. These items specifically addressed a) *the films' enhancement of tutors' preparedness to respond to specific challenges*, b) *the time frame allocated to the films*, and c) *the overall training concept including the films*. Phrases were again positively worded and rated using a visual analogue scale ranging from 0 = completely disagree to 100 = completely agree (see Table 1).

Questionnaires were returned by 101 of the109 participants (92.66%); 37 (36.63%) of the returnees were female and 64 (63.37%) male. Mean age was 34.04 ± 7.43 years. Professional experience ranged from 0 to 35 years with a median of 5 years.

Research was carried out in compliance with the Helsinki Declaration. In accordance with national practice in the country in which this study was carried out ethical approval is not required for such educational studies and surveys. We confirm that no plausible harm to participating individuals arises from the study and that participants cannot be identified from the presented material.

### Statistical analysis

Age and professional experience were assessed prior to training and data are presented as mean and standard deviation (age) or median and range (professional experience). To determine pre-post changes in the five items addressing self-efficacy with regard to PBL-related knowledge and skills, as well as attitudes, paired Student's *t*-tests were calculated. Results were adjusted using the Bonferroni approach. Statistical significance was considered for *p *< .05. Post-intervention ratings of *time frame*, *overall concept*, and *preparedness to respond to specific challenges *are presented as means and standard deviations.

## Results

We present an interactive online training tool for PBL tutors comprising 18 video clips highlighting major challenges most commonly encountered in PBL tutorials (see Table 2). Each video clip includes an initial sequence in which a specific tutorial situation is presented followed by an assignment to formulate own intervention strategies. In a second sequence, a suitable intervention is presented. Arguments in favour (*pro*) and against (*con*) the presented intervention and unresolved issues are subsequently discussed.

**Table 2:** Video clips of major obstacles encountered in PBL tutorials

Clip	Step	Content/problem
1	Prior to the tutorial	How to get the group started
2	Step 1, Present the case	Early activation of group members
3	Step 2, Define the problem(s)	Problem-definition: Phrasing the problem(s)
4	Step 3, Brainstorming (1)	A pause in brainstorming
5	Step 3, Brainstorming (2)	Early discussion arises within the brainstorming
6	Step 4, Generate hypothesis (1)	Splitting into small groups, a reflective tutor
7	Step 4, Generate hypothesis (2)	Splitting into small groups, an active tutor
8	Step 4, Generate hypothesis (3)	Competing threads of discussion
9	Step 4, Generate hypothesis (4)	Encouraging visualization
10	Step 4, Generate hypothesis (5)	Students directly addressing the tutor
11	Step 4, Generate hypothesis (6)	Quiet students, passive group
12	Step 5, Learning goals and Step 7, Synthesis (1)	Lack of commitment: objectives are not prepared
13	Step 7, Synthesis (2)	Diversion from the main topic, passive tutor
14	Step 7, Synthesis (3)	Diversion from the main topic, active tutor
15	Step 7, Synthesis (4)	Dominant tutor, constantly at the centre of attention
16	Step 7, Synthesis (5)	Disparaging comments within the group
17	Step 7, Synthesis (6)	Dominant student within the group
18	Concluding the tutorial	Successfully closing a tutorial

Video clips addressing major obstacles encountered in PBL tutorials. The seven steps of PBL and the content of the respective video clips are listed.

In the final part of this section we present data on acceptance, feasibility and self efficacy.

### Recommended integration of video clips into PBL tutor-training sessions

The following sequence is recommended for efficiently working with the videos in small groups or individually:

1) ***Presentation of the first sequence (film): ***The respective film clip commences with the presentation of a specific tutorial situation. The trainees are asked to carefully watch interactions between group members and the tutor. Note: the tutor is seated on the far left-hand side of the screen.

2) ***Assignment (text): ***In a text excerpt, trainees are assigned the task of developing their own intervention strategies: *What do you do now? *(Figure 1). Note: as a general rule, trainees should first decide whether they *need to intervene at all*, whether it is necessary *now*, and *how *the intervention should take place in order to foster cooperative and productive group processes.

3) ***Presentation of the second sequence (film): ***The video clip then continues and trainees are now confronted with a potentially suitable intervention. They are asked to watch the tutor intervene or not intervene and to examine the impact this has on the group process.

4) ***Discussion (text): ***In a second text excerpt, the intervention is recapitulated (*What was done*) and arguments in favour (*pro*) and against (*con*) the presented intervention are discussed in a concluding discussion. *Solutions *to unresolved issues in this situation are discussed. Finally, the *tasks for the tutor *in this situation are summarized (Figure 2).

### Clip 1: Get the group started, flashlight

It's the first session of your PBL tutorial. You have introduced yourself as the tutor of the upcoming tutorials for this group.

#### Presentation of the sequence

The tutor invites the group members to introduce themselves. The steps of PBL (seven-jump) are then clarified. Group members' expectations are collected and used to form a contract with respect to *being on time*, *everybody working on the learning goals*, group *conduct *etc.

#### Discussion

Pro: The presented scenario shows an ideal PBL group with respectful and appreciative interaction between participants. Con: Although this is a crucial situation for the beginning of successful tutorials, no time should be wasted on trivialities. Solution: The idea behind this introductory session is to allow prior positive as well as negative experiences in PBL tutorials to be mentioned and to empower group members to form an efficient learning group in which collaborate learning processes unfold and in which all members identify with the group. With a general remark, the tutor encourages everyone to speak out loud within the group and allows each member to express his/her opinion. The attitude to be conveyed is: *Each contribution is valuable*.

#### Tasks for the tutor

To lay the foundation for an efficient learning group.

### Clip 2: Step 1, Present the case and clarify terms

#### Presentation of the first sequence

The case to be discussed in this PBL tutorial is read aloud by a volunteer. The group hesitates to clarify potentially unfamiliar terms.

#### Assignment

What do you do now?

#### Presentation of the second sequence

The tutor asks *Are there any terms that are unclear? *at a rather early stage.

#### Discussion

Pro: The fact that the tutor takes an active role at such an early stage in the tutorial will speed up the initial step of the tutorial leaving more time for interaction in the following steps. Con: There was no need for such an early intervention. An early intervention at this point may cause attention in the following steps to be focused on the tutor. Solutions: Let the group elect a time-keeper to take over this part of structuring the tutorial. The tutor doesn't need to intervene this early but needs to some extent to tolerate pauses and silence in the group before intervening in order to facilitate active reflection upon the group process.

#### Tasks for the tutor

Early activation of group members. Not to intervene too early in order not to disencourage self-organization of the group.

### Clip 3: Step 2, Define problem(s)

The problem has been presented. Now the group proceeds to Step 2: *Define the problem(s)*.

#### Presentation of the first sequence

The group is efficiently self-organized and they thoroughly discuss the definition of the problem. The tutor does not intervene.

#### Discussion

Pro: The efficient self-organization and structuring within the group required no intervention on the part of the tutor. The spirit within the group was very good and the tasks involved in this step were accomplished. Con: The problem was defined using single terms (*carburettor*) instead of a phrase or sentence (*Why is the car not running?*). This may result in an unclear definition of the focus for subsequent brainstorming. The broad selection of very different aspects, such as mechanical and psychosocial or explanatory and procedural approaches, may lead to the upcoming session being overloaded: Can the group really address all of the issues in their problem definition? Solutions: Ask the group to formulate the problems in full phrases, to decide in favour of explanatory and/or procedural approaches, and to focus and reduce the problems for the initial session in order to leave room for further aspects if time permits.

#### Tasks for the tutor

To foster self-organization and structuring within the group and to ensure comprehensive but precise problem formulation.

### Clip 4: Step 3, Brainstorming (1)

The next step is the brainstorming phase in which all ideas relating to the problems defined in Step 2 are collected.

#### Presentation of the first sequence

Having been informed about the rules of brainstorming, the group successfully conducts this step. All group members are involved. Now there is a pause.

#### Assignment

What do you do now?

#### Presentation of the second sequence

The tutor hands back over to the group: Are there any more points?

#### Discussion

Pro: At this point, the tutor is patient and delays her intervention to encourage the groups' own efforts. More contributions are likely to be made at this stage without additional intervention. Con: If the brainstorming process is seriously stagnating refocusing the group may help. However, more active behaviour on the part of the tutor always carries the risk of attracting the group's attention. Solutions: Refer to the problem definitions or any diagrams generated so far: *Looking at the problems you have defined and the points you have made so far, are there any new aspects that come to your mind? *Keep in mind that if no further aspects are mentioned after such a prompt, then there is no point in pressing the group to produce more ideas at this moment in time. If major aspects have not been mentioned, keep them in mind to insert them at a convenient point in the ongoing tutorial. An alternative approach in the brainstorming phase is to have members independently and silently write items on cards, thus giving everyone, including more passive participants, a chance to contribute. The risk associated with this approach is a loss of the aspect of collective brain-*storming*.

#### Tasks for the tutor

To facilitate active and extensive brainstorming.

### Clip 5: Step 3, Brainstorming (2)

*Presentation of the first sequence: *A discussion has started between two participants within the brainstorming phase. The others remain passive.

#### Assignment

What do you do now?

#### Presentation of the second sequence

The tutor reminds the members to remain in the brainstorming phase and to refrain from discussing the issues in detail at this stage.

#### Discussion

Pro: Stopping the discussion at this stage is generally useful for ensuring that issues remain fresh and interesting for the upcoming discussion in Step 4. Additionally, not stopping the discussion may cause extensive brainstorming to be limited, since detailed discussion at this stage will narrow down the potential fields of discussion resulting in a loss of issues. Con: The tutor might put an end to a very productive discussion, losing valuable thoughts as well as motivation. Solutions: If a highly productive and valuable discussion arises straight away, then you could, *as an exception*, allow it to be continued at this point for limited issues and for a limited time only. It is necessary to clarify this for the members: *Now you have already begun discussing this issue. Let's go on for the moment just on this issue and then come back to the brainstorming*.

#### Tasks for the tutor

To postpone the discussion for the next step without inhibiting a highly productive discussion.

### Clip 6: Step 4, Generate hypothesis (1)

All issues have been discussed already and are now arranged to tentative solutions. Prior knowledge is activated and arising questions are addressed.

#### Presentation of the first sequence

An intense discussion is underway, but the group is split into small subgroups.

#### Assignment

What do you do now?

#### Presentation of the second sequence

Members of each small subgroup do not profit from the contents of the other subgroup discussions. The tutor asks the group to reflect upon the group process, leaving the group members themselves to define the communicative obstacle and to develop a solution to this problem.

#### Discussion

Pro: The intervention aims to make the discussion more effective by drawing together all members. Con: Although the group needs to be refocused at this stage for efficient group work, an intervention bears the danger of restraining or even impeding group processes as well as restricting the activity of the members. Solutions: Groups of more than 10 participants are particularly at risk of forming such small-group discussions. Any intervention should aim to show appreciation for the dedication and the input of group members on the one hand, at the same time as merging the various threads of discussion on the other. The group can subsequently prioritize and decide what to tie in first.

#### Tasks for the tutor

To promote group members' reflection upon the group process involved in the subgroup discussions and to merge the various threads.

### Clip 7: Step 4, Generate hypothesis (2)

Again, issues are being discussed. Prior knowledge is activated and arising questions are addressed.

#### Presentation of the first sequence

The discussion is just as intense and is again taking place in small groups.

#### Assignment

What do you do now?

#### Presentation of the second sequence

Again, a very intense discussion was underway and the group was split into small subgroups so that the contents of small-group discussions were lost for the others. The tutor asks group members to focus on one topic at a time as opposed to inviting them to reflect upon the group process as in the prior clip.

#### Discussion

Pro: This will enable a more effective discussion in which all members can take part. Con: The tutor did not prompt group members to reflect on the process (as seen in Clip 6) and thus steered the group in a more explicit manner. In general, steering will always attract attention to the tutor and leave the group more passive in upcoming difficult situations. Solutions: The tutor could ask the group to reflect on the process. As seen in Clip 6, an intervention should also aim to demonstrate appreciation for the dedication and input of group members.

#### Tasks for the tutor

To help the group reflect upon the group process and refocus.

### Clip 8: Step 4, Generate hypothesis (3)

The discussion continues.

#### Presentation of the first sequence

Different threads of discussion are brought up by the group members.

#### Assignment

What do you do now?

#### Presentation of the second sequence

The tutor refocuses the discussion.

#### Discussion

Pro: The tutor's refocusing of the discussion helps the group to make progress. She leaves the decision regarding which topic should be focused on first to the group. Con: The tutor should not allow her intervention to cause new and interesting ideas to get lost. Solutions: As a tutor, it is essential that the ideas brought up by group members are respected and valued. At the same time, however, effective progress necessitates that some issues need to be and may easily be postponed: *This is a very good idea. Let's keep this in mind and come back to this later*. A further option is to let the group rank the issues which they would like to dwell on first.

#### Tasks for the tutor

To value ideas brought up by group members and facilitate focusing and prioritizing within the group.

### Clip 9: Step 4, Generate hypothesis (4)

The group is in the process of discussion.

#### Presentation of the first sequence

One group member announces: *I have a problem visualizing this*.

#### Assignment

What do you do now?

#### Presentation of the second sequence

The tutor rightly decides not to intervene. The group was - at least in the beginning - active. One group member subsequently starts to hold a lecture; the tutor still does not intervene.

#### Discussion

Pro: It is important for the group members to visualize their ideas. The tutor should encourage visualization but also ensure that the flow of activity is not hindered. Visualization fosters questions that otherwise would not arise and that contribute to a deeper understanding. Con: Visualization at this point is primarily performed by only one group member; two further members remain passive (boy in blue shirt on the left and girl in black blouse on the right). Solutions: If the group is too focused on a flipchart, place the flipchart paper at the centre of the table and ask everyone to join in. Make sure that visualization takes place at all within the group. Possible interventions for the fostering of visualization could be: *What still remains unclear to you? How would your ideas so far fit into a diagram? *Incorporate the two passive members: *You don't look very convinced at the moment. What remains unclear to you? *Refer to the lecturing of the one member at the end of the session or ask others to join in: *Thank you, that is absolutely right. But let's have the others join in: What is still unclear to the others? Who else can explain?*

#### Tasks for the tutor

To encourage visualization and incorporate passive members.

### Clip 10: Step 4, Generate hypothesis (5)

We proceed in the discussion.

#### Presentation of the first sequence

During the discussion, several questions are directed towards the tutor.

#### Assignment

What do you do now?

#### Presentation of the second sequence

The tutor responds to the first question by passing it back to the group. She responds to the second question with which she is addressed by referring to the diagram created by the group. After each intervention, she leans back and allows group activity to unfold.

#### Discussion

Pro: The tutor gives the question back to the group at a point where she expects that they will be able to find a solution and thus keeps the group discussion going. Con: If simple questions arise which the group is not able to answer straight away, the tutor may provide this small piece of information in order to encourage continued discussion at a higher level. A classical lecture provided by the tutor would, however, represent a threat to the group process. Solutions: Any intervention (including the input of small pieces of information) should aim to restart the group process with a question which leads to discussion at a higher level. Typical questions include: *Does this change your explanations/ideas so far? What can you do now? Can you give reasons? *You may refer to the items and/or diagrams which have so far been generated by the group by pointing out unclear issues. Watch the tutor lean forward, placing herself at the centre of attention. By leaning back after her intervention, she once again removes herself from the focus of attention, as a result of which, the group members will automatically refocus on themselves.

#### Tasks for the tutor

To restart the group process with a question which leads to discussion at a higher level. To (literally) withdraw him/herself from the focus of attention after the intervention.

### Clip 11: Step 4, Generate hypothesis (6)

The group is still in the process of a discussion, which is beginning to ebb away.

#### Presentation of the first sequence

The group process is coming to a halt. No one comes up with a new issue.

#### Assignment

What do you do now?

#### Presentation of the second sequence

The tutor has more patience than most would have but lets the group remain silent for just 50 seconds.

#### Discussion

Pro: The tutor shouldn't allow attention to be drawn to his/her own person too quickly. Con: On the other hand, the tutor must avoid tutorials which are inefficient and boring and the goals of the tutorial need to be achieved. Allowing the silence to extend over too long a period may mean a considerable loss of fun and motivation. Solutions: Refer to prior diagrams or key points on the flipchart or whiteboard to reactivate the prior discussion. You may ask questions like: *Why ....? Why not ...? What next? *etc. Alternatively, a group member might sum up the results achieved so far.

#### Tasks for the tutor

Not to allow the group to focus on the tutor too quickly. To refer to prior diagrams or key points on the flipchart or whiteboard in order to reactivate the prior discussion.

### Clip 12: Step 5, Learning goals and Step 7, Synthesis (1)

After the self-study (step 6) concerning the learning goals and objectives formulated on day one, the session on day two commences with a discussion on the results of the self study (step 7). The tutor asks the group to address the learning objectives of the prior session.

#### Presentation of the first sequence

The group is obviously ill-prepared.

#### Assignment

What do you do now?

#### Presentation of the second sequence

The tutor assures herself that no one has worked on the learning goals before pointing out how ineffective the group's learning process will be if learning goals are not worked on.

#### Discussion

Pro: At this point the tutor needs to be very clear. There is no point in continuing the discussion from where it was stopped on day one (step 5) if the group didn't work on the learning objectives in the self-study (step 6). If nothing has been learnt, a discussion on a higher level cannot develop. This is a critical point for a successful PBL tutorial and makes for the difference of a PBL tutorial and a classical lecture. Con: None. There is no need for lecturing by the tutor at this point since this would only further disencourage own activity in the future sessions in Step 6. Solutions: clarify who has prepared the learning goals and who has not. This problem of group members not being prepared can be alleviated by making a clear contract at the beginning of the tutorials (see Clip 1, get the group started). At this point, the tutor needs to help the group reflect on the reasons why they did not prepare the learning matter. *Are they not relevant? Are they too easy or too complex? Were the learning goals imposed on them by the tutor in Step 5? Are they considered irrelevant to future assessments?*

#### Tasks for the tutor

To clearly point out the ineffectiveness of the group's learning process when learning goals are not prepared.

### Clip 13: Step 7, Synthesis (2)

The group continues the discussion.

#### Presentation of the first sequence

In the course of the discussion, one group member diverts to another topic.

#### Assignment

What do you do now?

#### Presentation of the second sequence

The tutor does not intervene at this point.

#### Discussion

Pro: The group has already taken care of the diversion. Con: Good and new ideas could be lost in the process of topic diversion. Solutions: If the group adequately takes care of irritations caused by single members, then there is no need for intervention by the tutor. Make sure important or valuable new ideas and relevant threads of discussion do not get lost in the process of the group agreeing on issues for discussion.

#### Tasks for the tutor

Not to intervene if the group takes care of irritations caused by single members in an adequate way.

### Clip 14: Step 7, Synthesis (3)

Still in the discussion, a further irritation occurs.

#### Presentation of the first sequence

Again, a single group member diverts to another topic which is far removed from the issues which were being discussed.

#### Assignment

What do you do now?

#### Presentation of the second sequence

The tutor now intervenes (in contrast to Clip 13) and then refers back to the group.

#### Discussion

Pro: The tutor refocuses the group on the central topic of their discussion. Con: Diversions from the topic can also represent creative impulses which help to make a tutorial both interesting and humorous. Solutions: (Almost) all contributions carry some value. Interventions by the tutor should thus be accompanied by an appreciation of the individual contributions. Nevertheless, it is necessary to help the group prioritize what to deal with and what not in order to encourage them to refocus. You could ask questions like: *Looking at the diagram, you were currently trying to explain one issue. Why don't you focus on this for the moment and come to the other aspects later? *Or: *This is a new issue and will require a lot of discussion. Do you want to focus on this new issue now?*

#### Tasks for the tutor

To refocus the group at the same time as showing appreciation for each individual contribution.

### Clip 15: Step 7, Synthesis (4)

The group again is in a phase of discussion

#### Presentation of the sequence

All questions are addressed to the tutor. The tutor immediately answers these questions as a result of which participants are encouraged to address even more questions to her. Note her position at the centre of the group's focus underlining her directive role.

#### Discussion

Pro: There is actually little to be said in favour of such an over-motivated style of 'taking over' a PBL tutorial. Con: The tutor's activity destroys productive and constructive group work. Although this example is exaggerated, it indicates the constant risk with which the tutor is faced. Solutions: As seen in Clip 9, any intervention should aim to restart the group process with a question which leads to discussion at a higher level. Typical questions are: *Does that change your explanation or ideas so far? What can you do now? *You may refer to the visualizations of the group which have been made so far. The first step is to lean back after your intervention in order to keep the focus within the group.

#### Tasks for the tutor

To restart and activate the group process. To refrain from lecturing.

### Clip 16: Step 7, Synthesis (5)

The discussion continues.

#### Presentation of the first sequence

One member has offended another.

#### Assignment

What do you do now?

#### Presentation of the second sequence

The tutor intervenes to pacify the two participants.

#### Discussion

Pro: Insults require immediate intervention provided that the group members do not intervene themselves. But be fair and impartial in your intervention. Con: Objective interventions means appreciating both sides including the potential intention of the offender! Solutions: You must remain impartial and fair. You may refer to the initial contract made by group members (see Clip 1, get the group started). Being objective means not taking sides. The insult must on the one hand be stopped. On the other hand, the insulting intervention may, although intolerable in its form of presentation, stem from a justified point of criticism. Your role is to translate the offensive insult into its justified meaning, if the group members are unable to do so themselves. In this clip, the offender's message may be translated to: *Shouldn't we all prepare our learning goals to make this group work successfully?*

#### Tasks for the tutor

To intervene if the group members do not do so themselves. To stay fair and not to take sides. To translate an offence into the intended and potentially justified criticism.

### Clip 17: Step 7, Synthesis (6)

Discussion continues.

#### Presentation of the first sequence

An eager participant explains a new aspect with a lot of valuable details but too fast for the group to follow. The group remains passive.

#### Assignment

What do you do now?

#### Presentation of the second sequence

An ambitious participant explains a new aspect while the group remains passive. This participant shares thorough and rapid information which is, however, lost because of the speed at which it is conveyed; the rest of the group becomes increasingly passive and there is no collaborative process. There is no attempt from the group to stop the participant. The tutor then asks the participant to visualize what he has just explained.

#### Discussion

Pro: In asking the participant to visualize, the tutor shows appreciation for the knowledge shared at the same time as reducing the speed of communication and fostering understanding within the group so that questions can arise. This should allow the group to catch up and be reintegrated. Con: Take care not to have the expert participant lecture for too long a period in case the group once again becomes passive. Solutions: Show appreciation for the ideas of the expert participant. Actively include him in the process, by for example, having him visualize the content, but prevent one-to-one communication and lecturing. This can be done by encouraging "why" questions or: *What is still unclear? Can anybody else relate that to what we discussed earlier on*?

#### Tasks for the tutor

To show appreciation for the expert participant. Actively include him in the process but prevent one-to-one communication and lecturing.

### Clip 18: Step 7, Concluding the tutorial

We have reached the end of a successful tutorial.

#### Presentation of the sequence

The tutor remains rather inactive and absent-minded. The group functions well and is well-organized

#### Discussion

Pro: There are not really any pros. Con: This may serve as a negative example of a careless tutor not paying attention or showing appreciation. It may also show how well a committed group can function without a tutor. Solutions: We would like to encourage *attentive preparedness *of the tutor: The tutor should be aware of the group process but not intervene if the group is able to take care of things for themselves. Before you intervene, consider 1) whether you really need to intervene, 2) whether this is the right time to do so, and 3) how the intervention might foster a cooperative and productive group process.

#### Tasks for the tutor

We would like to encourage *attentive preparedness *of the tutor; the tutor should be aware of the group process.

### Acceptance, feasibility, and perceived effect of the video clips

Of the N = 109 staff Heidelberg Medical Faculty participated in our PBL tutor-training as described in the methods section. Of these 36.63% were female and 63.37% male, mean age was 34.04 ± 7.43 years and professional experience ranged from 0 to 35 years with a median of 5 years.

Pre-post comparisons of tutors' ratings revealed that the entire training courses including the interactive training tool led to an increased understanding of PBL as a method (pre: 48.80 ± 29.28, post: 81.92 ± 16.00; p < .0001) and the role of a tutor in PBL (pre: 46.11 ± 28.89, post: 81.16 ± 15.51; p < .0001). Participants felt that the training had improved their preparation for PBL tutoring (pre: 34.46 ± 26.76, post: 70.28 ± 20.08; p < .0001) and their ability to face problematic situations as a tutor (pre: 36.47 ± 26.25, post: 66.99 ± 21.01; p < .0001). The training significantly increased appreciation of PBL as a didactic method (pre: 61.33 ± 24.84, post: 76.20 ± 20.12; p < .0001; all ratings on visual analogue scales ranged from 100 = completely agree to 0 = don't agree at all). Differences between pre- and post-intervention self-efficacy ratings remained significant after conservative Bonferroni adjustment for five statistical tests.

After training with the interactive training tool, trainees considered the video clips to have prepared them well for responding to specific challenges (75.98 ± 19.46) and the time spent on the films to have been adequate (62.94 ± 16.76). The overall training concept including the films was rated highly (81.62 ± 16.91; all ratings on visual analogue scales ranged from 100 = completely agree to 0 = don't agree at all; see Table 1).

## Discussion

Given the significant effects of PBL tutor-training programmes on faculty and medical education programmes, surprisingly little data has been published on the details of such training schedules or the employed training material. To our knowledge, this is the first publication to report on a PBL tutor-training course designed to foster tutors' expertise in handling specific problems commonly encountered in a PBL tutorial.

In this study, we present an interactive online training tool for PBL tutors and describe its successful integration in the PBL tutor-training that has regularly been offered to and attended by most of the novice staff involved in PBL tutoring at our Medical Faculty since 2003 [2,35] (Figure 1, Figure 2). Over the past seven years, we have trained more than 600 PBL tutors at our faculty and participants rate the overall concept as being very successful. Owing to the heterogeneous background of the medical experience of our participants (ranging from junior house officers to consultants), the underlying case applied in our training courses is non-medical; we are thus able to improve their interest and contributions irrespective of prior medical knowledge [2]. As shown in the present study, the alignment of the non-medical case and our training tool is very well accepted. Furthermore, the entire training led to higher self-efficacy ratings regarding PBL-related knowledge and skills as well as higher ratings with respect to tutors' attitudes towards the method. The presented interactive online training tool for PBL tutors comprises a set of 18 films addressing problems arising in PBL tutorials and invites tutors to reflect upon potential intervention strategies. Educational films have continuously gained in importance in medical education over the last 50 years [36], and the integration of video technology has become part of the mainstream in postgraduate medical-education [37]. However, very little has been published on the integration of videos in PBL tutorials [38,39], and we are not aware of any reports on the perceived effects of videos in PBL tutor-training. In the context of undergraduate medical-training in Maastricht, the integration of videos in student PBL-tutorials has been shown to be effective: The presented videos challenged students to elaborate more extensively upon the cases, which also proved to be more memorable as compared with purely text-based cases [38]. Similarly, the integration of videos in postgraduate PBL-tutorials has been found to improve cognitive and metacognitive processes, such as theory building and theory evaluation, among residents [39]. Our tool may be used as a role play and thus offers opportunities to foster such cognitive and metacognitive processes among PBL tutors in the setting of PBL tutor-training: It allows novice tutors to reflect upon their verbalization and paraphrasing of specific interventions in response to threats to optimal group functioning. In our study, the tool improved participants' understanding of the tutor's role. Consistent with the collaborative approach in PBL, tutors can also use the video sequences to develop solutions to the presented problems in a collaborative fashion, thus providing tutors with feedback on their interventions in a protective environment. Tutors may develop and reflect upon their role as a professional facilitator in a stepwise manner. The clips may serve as an introduction to the role as tutor and to appropriate approaches for moderating a tutorial for novice tutors as well as spark discussions based on the specific scenarios as a form of continuous training for more advanced tutors.

Our films improve participants' preparedness for their role as tutor; a finding which may, at least in part, be due to critical appraisals of the model tutor in the presented films. The general need for and potential impact of positive role modelling as a teaching tool is unquestioned [40]. Tutors involved in PBL programmes must undergo a change of attitude in order to enable them to engage in successful facilitation, and novice tutors thus benefit from the experiences of more experienced tutors [41]. The tutor in our video clips serves as a role model with a greater degree of authenticity than can be achieved by a paper-based manual. In particular, strategies for role modelling and non-verbal communication are essential for facilitation [30] but hard to put into practice when conveyed in a text-based format. These arguments have strong implications for the design of tutor-training programmes [41]: We consider role modelling in PBL tutor-training programmes to be essential for the development of a sustainable change in the skills and attitudes of novices. In this respect, our video clips constitute a valuable supplementary tool for tutor training.

The employed video clips cover a wide range of problematic situations which can potentially threaten productive group work in PBL tutorials. While previous publications have summarized the relevant problems arising in PBL tutorials [24,27], they have failed to offer specific approaches regarding how tutors should handle them. In a first step, our videos aim to increase tutors' awareness of such critical situations and to promote professionalization with respect to the timing of interventions and the development of own intervention strategies. In a second step, the discussion of the pros and cons which is integrated in our tool allows the risks and benefits associated with specific interventions to be weighed up as a basis for successful facilitation. In line with the finding that the integration of videos increases confidence in trained skills in postgraduate medical-training [42], the videos in our study enhanced tutors' confidence in their facilitation skills. Tutor confidence has, in turn, been shown to have a significant positive impact on students' learning and satisfaction as well as on their attainments in final examinations [8,43]. As an additional effect of PBL tutor-training programmes, the training of specific teaching skills has been found to result in an increase in active curriculum-participation on the part of the trained staff [44] and thus represents a key to successfully integrating PBL into a curriculum [8,22]. The observed increase in positivity of attitudes towards PBL following training with our tool underscores this potential.

In transitioning to their new role as facilitator, novice tutors require training which equips them to manage the *specific *challenges with which they will be faced in the teaching-learning process [41]. Training with our tool enhances tutors' confidence in tackling these specific problems by encouraging them to first decide whether or not to intervene and to subsequently develop adequate individual intervention strategies. Decisions regarding *when *and *how *to intervene in the PBL process are critical for successful PBL tutoring [30] and should therefore constitute a primary focus of any PBL tutor-training programme. Hendry et al. found tutor interventions to be least effective in the face of *quiet *or *dominant students*, *a lack of commitment*, and *bullying *or *disparaging of students *on the part of students [27]; problems which are all addressed by our tool. Due to our decision to focus on group processes within an ongoing PBL tutorial, we did not include Hendry et al.'s item *lateness of individuals *[27]; we considered the occasional lateness of individuals - while disturbing - to be rather unspecific to PBL tutorials. In the present study, training these specific scenarios was found to increase participants' self-efficacy with respect to facing such challenges in their future tutorials.

Training with our videos (analogous to the '*shows how' *in Miller's pyramid [45]) cannot replace the indispensable solid training and supervision of future PBL tutors in a 'real' setting ('*does' of the *Miller's pyramid [45]). However, these videos are intended to bridge the gap between PBL tutor-training in an artificial, didactically framed setting and encounters with challenging situations in the real-life context of the PBL process. The presented tool is accessible online at http://www.medizinische-fakultaet-hd.uni-heidelberg.de/fileadmin/PBLTutorTraining/player.swf (Figure 2) and can be readily integrated into a training course or used as personal preparation or a refresher at home.

### Implications for future research

The presented tool can be used to study the added value and effect of videos on PBL-tutor performance, that is, on students' ratings of tutors' skills and attitudes, as well on the sustainability of such training effects. The video clips also offer the opportunity to investigate the impact of videos on the cognitive and metacognitive processes of PBL tutors. Further investigations should address these quantitative and qualitative issues in a controlled study.

## Conclusions

We present a well accepted and feasible tool for PBL tutor-training which represents a *technical advancement *to be shared with the medical-education community. The tool comprises an extensive set of difficult situations that PBL tutors may face and, as is shown, can be successfully integrated into PBL tutor-training. The tool aims to sensitize PBL tutors to situations which pose a potential threat to successful group work and to help tutors develop individual strategies which foster productive learning processes.

The integration of video-based scenarios of critical situations represents a valuable PBL-training approach which can be adopted by medical faculties to train both novices and experienced tutors. Novice PBL tutors can be made aware of potential challenges to successful group work and develop professional intervention strategies promoting a productive learning process. For expert tutors, the tool can help spark discussions regarding the pros and cons of interventions based on the specific scenarios presented. We would like to share this tool with the medical-education community to bridge the gap between PBL tutor-training in an artificial, didactically framed setting and encounters with challenging situations in the real-life context of PBL tutorials.

## Competing interests

The authors declare that they have no competing interests.

## Authors' contributions

All authors participated in the planning and design of the study. All authors read and approved the final manuscript. HMB and SS wrote the screenplays. CN organized actors and technical instrumentation for the film shooting, HMB, SH, SS, and CN supervised the shooting of the films. HMB and CN wrote the manuscript. MK organized the PBL training courses. CN organized fundraising by the Medical Faculty of Heidelberg to support these films.

## Pre-publication history

The pre-publication history for this paper can be accessed here:

http://www.biomedcentral.com/1472-6920/10/52/prepub
